# Factors affecting antenatal care attendance in Soweto, Johannesburg: The three-delay model

**DOI:** 10.4102/phcfm.v16i1.4333

**Published:** 2024-06-14

**Authors:** Nellie Myburgh, Thabisile Qwabi, Lunghile Shivambo, Lerato Ntsie, Andile Sokani, Maria Maixenchs, Isaac Choge, Sana Mahtab, Ziyaad Dangor, Shabir Madhi

**Affiliations:** 1 Department of Vaccines and Infectious Disease Analytics, Faculty of Health Sciences, University of the Witwatersrand, Johannesburg, South Africa; 2 Barcelona Institute for Global Health Hospital Clínic, Faculty of Health Sciences, Universitat de Barcelona, Barcelona, Spain

**Keywords:** antenatal care attendance, women, pregnancy, maternal mortality, infant mortality, public clinics, three-delay model

## Abstract

**Background:**

Antenatal care remains critical for identifying and managing complications contributing to maternal and infant mortality, yet attendance among women in South Africa persists as a challenge.

**Aim:**

This study aimed to understand the challenges faced by women attending antenatal care in Soweto, Johannesburg, using the three-delay model.

**Setting:**

This study was conducted in Soweto, Johannesburg.

**Methods:**

An exploratory, descriptive and qualitative research design was used, and in-depth interviews were conducted with 10 pregnant women and four women who had recently given birth.

**Results:**

Findings indicate delays in seeking care due to factors such as pregnancy unawareness, waiting for visible signs, and fear of human immunodeficiency virus (HIV) testing. Challenges such as transportation difficulties, distance to clinics, and facility conditions further impeded the initiation of antenatal care. Late initiation often occurred to avoid long waits, inadequate facilities, language barriers and nurse mistreatment.

**Conclusion:**

From this study, we learn that challenges such as unawareness of pregnancy, cultural notions of keeping pregnancy a secret, fear of HIV testing, long waiting lines, high cost of transportation fees, clinic demarcation, shortage of essential medicines, broken toilets and verbal abuse from nurses have delayed women from initiating antenatal care early in Soweto, Johannesburg.

**Contribution:**

Challenges of women with antenatal care attendance in South Africa must be addressed by implementing community-based health education interventions, institutionalising HIV psycho-social support services and improving quality of antenatal care services in public health facilities.

## Introduction

The World Health Organization (WHO) recommends that pregnant women must attend a minimum of eight antenatal care contacts.^1,2,3^Antenatal care contacts help health care professionals identify problems that could pose risk to the health of the mother and the baby.^4^ Interventions such as micronutrient supplementation, treatment for hypertension to prevent eclampsia and tetanus immunisation are provided to pregnant women.^5,6^ Maternal infections such as human immunodeficiency virus (HIV) and syphilis are detected and treated to prevent mother-to-child transmission that could lead to premature births, low birth, stillbirths and neonatal deaths.^7,8,9,10^ South Africa has adopted the WHO recommendations through the Basic Antenatal Care (BANC) policy, and essential antenatal care services are offered for free in all public health facilities.^10,11,12^ Women are encouraged to attend their first antenatal care visit before 20 weeks of gestation.^13^ However, women in South Africa only start attending antenatal care after 20 weeks of gestation, and in some cases, they do not attend at all.^13,14^ In 2020, only 68.3% of women in South Africa attended their first antenatal care visit before 20 weeks of gestation, and this was a decline from the 69.7% recorded in 2019 (Table 1).^14^

**TABLE 1:** Antenatal care first visit before 20 weeks of gestation.

Province	2018 (%)	2019 (%)	2020 (%)
Western Cape	70.2	71.7	70.7
Eastern Cape	62.4	63.4	63.2
Northern Cape	63.2	65.6	60.7
Free State	65.2	66.6	62.2
KwaZulu-Natal	72.8	74.4	74.6
North West	68.4	71.3	68.1
Gauteng	64.0	66.4	63.9
Mpumalanga	75.2	77.0	75.1
Limpopo	66.8	68.8	67.5
South Africa	67.8	69.7	68.3

*Source*: Makua M, Massyn N. Antenatal 1st visit coverage. In: Massyn N, Day C, Ndlovu N, Padayachee T, editors. District health barometer 2019/20. Durban: Health Systems Trust; 2020

The low uptake of antenatal care services before 20 weeks of gestation in South Africa poses a public health risk and contributes to preventable maternal and infant deaths.^10,14,15^ In 2020, the maternal mortality ratio at the facility level was 88.8 per 100 000 live births, the neonatal mortality ratio was 11.9 per 1000 live births and the stillbirth rate was 19 per 1000 live births.^14,15^ The leading causes of infant and maternal deaths, such as non-pregnancy related infections, obstetric haemorrhage, hypertensive disorders, intrauterine hypoxia, low birth weight and sepsis could be detected, prevented and treated during antenatal care attendance.^16^ In a study by Hoque et al.^17^ in KwaZulu-Natal, pregnant women who presented late or did not attend antenatal care were more likely to experience adverse birth outcomes such as prematurity, low birth weight, stillbirth and neonatal deaths.

Challenges associated with women’s late presentation for antenatal care in South Africa include insufficient knowledge about antenatal care, pregnancy not being viewed as a life-threatening condition and cultural beliefs about the dangers of early pregnancy disclosure.^18,19^ The age of the mother, literacy level, access to quality health care services, long distance to health facilities, affordability of transportation, poverty and unemployment have been associated with women’s late presentation to antenatal care.^20,21,22,23^ Women who had uncomplicated pregnancies in the past do not perceive antenatal care visits as essential.^24^ Unplanned pregnancies, especially among teenagers were associated with low uptake of antenatal care service.^25^ Women who were married were more likely to initiate antenatal care compared to unmarried women.^26^ According to Horner et al.,^27^ employed and unemployed women in South Africa present late for antenatal care. This contradicts studies from other parts of the world which show that women who are employed tend to present late for antenatal care because of their busy schedules.^28^ In communities where there is a high prevalence of HIV, women reported that they feared attending antenatal care because the nurses were going to test them for HIV.^29^ The HIV Prevention-of-Mother-to-Child intervention has been successful in treating maternal HIV diagnosis to ensure that the baby is not exposed to HIV.^29^ However, the downside is that women must deal with the psychological burden of an HIV diagnosis during pregnancy.^30^ In South Africa, some cultural groups view pregnancy as a sacred rite of passage, and it should be kept as a secret from the public eye.^31^ Attending antenatal care before 3 months is prohibited as it will be a form of publicising the woman’s pregnancy to the community thus, putting the woman and the unborn baby at risk of spiritual attacks.^31^ The aim of this study is to understand the challenges of women with antenatal care attendance in Soweto, Johannesburg by revisiting the three-delay model.

### Conceptual framework: Three delays model

In 1994, Thaddeus and Maine^32,33,34^ theorised that three delays hinder women with obstetric complications from receiving lifesaving medical interventions in developing countries. The first delay is the decision to seek care during pregnancy.^32^ The woman’s decision to seek care during pregnancy is influenced by socioeconomic status, power dynamics within the family, indigenous reproductive health practices, perceived barriers to medical care and past experiences with health facilities.^32^ Women without formal education struggle to access reproductive health education about the significance of seeking medical care to avoid complications during pregnancy.^35^ Women from poor economic backgrounds struggle to seek care during pregnancy because they are subjected to long working hours within the informal sector and overburdened with unpaid caregiving at home.^35^ However, Thaddeus and Maine^32^ warn that in developing countries, even when a woman is in good economic standing, her decision to seek care during pregnancy is influenced by the power dynamics of the family. In these kinship structures, husbands, older women and mothers-in-law have authority over reproductive health decisions.^32^ Indigenous reproduction systems in developing countries perceive pregnancy as a normal development stage that does not require medical interventions.^36^ According to Thaddeus and Maine,^32^ even when a woman is experiencing an obstetric complication requiring urgent medical intervention, she first consults with the traditional healer, who decides whether she should proceed to the health facility. Perceived barriers such as cost of transportation, medical consultation fees and past experiences with quality of care provided at the health facility delay the woman’s decision to seek care during pregnancy.^36^

Once a pregnant woman and her family decide to seek care, they are met with the second delay, which occurs when trying to reach the health facility.^33^ In developing countries, reaching a health facility during pregnancy depends on the conditions of roads, availability and affordability of transportation, and distribution of health facilities.^34^ In rural areas, poor roads have led to women with obstetric complications dying on their way to the health facility.^37^ Even when roads are in good condition, reliable transportation, and the woman’s ability to afford transportation delay her from reaching the health facility on time to receive care.^37^ In developing countries, health facilities are concentrated in urban areas, leaving rural communities to rely on one health facility, often far away from where people live.^38^ Because of the uneven distribution of health facilities, pregnant women travel long distances to reach the health facility.^38^ When the family reaches the health facility, it would be too late to administer medical interventions to save the mother and the baby.^39^ According to Salih and Eltyeb,^39^ in developing countries, poor infrastructure, affordability and accessibility of transportation are structural determinants of health that contribute significantly to preventable infant and maternal deaths.

The third delay is the quality of health care services the pregnant woman receives once she reaches the facility.^40^ The provision of quality services during an obstetric complication depends on the availability of skilled health care workers, medical suppliers, medicines and efficient referral systems.^40^ In developing countries, when a woman with an obstetric complication reaches the health facility, there is no guarantee that she will receive lifesaving medical intervention on time.^41^ Pregnant women are subjected to long waiting times to receive care because health facilities lack enough human resources.^41^ Even when few health human resources are available, they are not skilled enough to deal with obstetric complications.^42^ Sometimes essential medicines and medical supplies such as anaesthesia necessary for emergency caesarean section would not be available.^42^ According to Harrison et al.,^43^ pregnant women are subjected to obstetric violence, where health care professionals verbally, emotionally and physically abuse them. Preventing maternal and infant mortality in developing countries requires improving the delivery of health care services in facilities. The thee-delay model in this study will help us to analyse ways in which personal, cultural, socioeconomic and health systems challenges influence women’s decision to attend antenatal care in Soweto, Johannesburg.

## Research methods and design

### Study design

An exploratory, descriptive and qualitative research design which captures the complexity of a social phenomenon by understanding the experiences of those affected by it was adopted in this study. Researchers applied an exploratory descriptive approach by having prolonged and detailed engagements with pregnant women and women who had recently given birth about their challenges with antenatal care attendance in Soweto, Johannesburg.

### Setting

This study was conducted in Soweto, which is in the city of Johannesburg, Gauteng Province, South Africa. Soweto is in Region D of the City, and it is home to 1 895 921 people where the majority are black people.^31^ Common languages that are spoken in Soweto are isiZulu, isiXhosa, Sesotho, Xitsonga and Setswana interchangeably.^31^ People in Soweto struggle with unemployment, poverty and burden of diseases such as HIV and acquired immune deficiency syndrome (AIDS), tuberculosis, hypertension and diabetes. Gauteng Province where Soweto is located has the highest number of maternal and infant mortality. In 2020–2021, the number of maternal deaths recorded in health facilities was 291, the number of stillbirths was 5143 and the number of neonatal deaths was 2266.^16^ To respond to the challenges of infant mortality in South Africa, the Child Health and Mortality Prevention Surveillance (CHAMPS) network implements medical interventions, scientific research and community-based programmes.^44,45^ The CHAMPS programme operates in Bangladesh, Ethiopia, Kenya, Mali, Mozambique, Sierra Leone and South Africa.^46,47^ The CHAMPS Social and Behavioural Sciences team at the University of Witwatersrand Vaccines and Infectious Disease Analytics (WITS-VIDA) conducted this study to understand the challenges of women with antenatal care attendance in Soweto, Johannesburg.

### Study population, sample size and sampling strategy

A total of 14 participants which included 10 pregnant women and four women who had recently given birth in the past 6 months were recruited to participate in this study. Researchers purposively sampled pregnant women and women who had recently given birth in the last 6 months because they had in-depth knowledge and experiences of antenatal care services in Soweto. Women with children who are older than 6 months were excluded from the study to reduce memory bias. Researchers recruited participants in seven communities in Soweto, namely Phiri, Emndeni, Meadowlands, Zone 5, Freedom Park, Mapetla, Jackson and Thembelihle. These areas were selected as they are part of the catchment area for the CHAMPS programme in Soweto, Johannesburg.

### Data collection

We utilised in-depth interviews to gather information regarding the experiences and obstacles encountered by women attending antenatal care. In-depth interviews were conducted by trained qualitative researchers in the home of participants from September to December 2022. The duration of the interviews was 40–60 min. Local languages such as isiZulu, Sesotho, Xitsonga and Setswana were used to conduct the interviews. All in-depth interviews were recorded after obtaining informed consent to do so from the participants.

### Data analysis

Thematic data analysis was used which allowed researchers to read transcripts, generate codes, take note of reoccurring themes and address the research objectives.^30^ The in-depth interviews were transcribed verbatim and translated into English before data analysis was conducted. After the in-depth interviews were transcribed, researchers read and re-read each transcript, identified re-occurring themes and developed codes. To ensure trustworthiness and validity of the data, all researchers were involved in analysing the data and checked language errors that would have occurred during translation. A data analysis software called Dedoose version 9.0 was used to assist in the process of organising themes from the data.

### Ethical considerations

Ethical clearance for this study was obtained from the University of the Witwatersrand Human Research Ethics Council (Wits HREC) and the approval number is 170216. Participants were informed that the aim of the study is to understand women’s challenges with antenatal care attendance in Soweto, Johannesburg. It was explained to participants that participation is voluntary and that they could withdraw from the study at anytime. They were also informed that the data they provide in this study will be used to generate research knowledge, produce publications and inform policy decisions about strengthening antenatal care services in Soweto, Johannesburg. The data participants shared were not linked to their personal information to ensure confidentiality. Personal information such as names or identifiers was omitted from the research findings to protect the identity of participants and ensure anonymity.

## Results

### Participants’ demographics

A total number of 14 participants agreed to participate in this study. When the data were collected, 10 women were pregnant, and four had recently given birth. Participants were between the ages of 21–40 years. The highest qualification obtained was high school education; most participants were unemployed. Marital status shows that most participants were not married but in relationships and cohabitating with their partners (Table 2). Parity data reflects seven participants were first-time mothers and the other seven had children before.

**TABLE 2:** Participants’ socio-demographics.

Unique identifier	Age	Parity	Relationship status	Employment	Education
Pregnant Woman 1-8M	22	1	In a relationship	Unemployed	High school
Pregnant Woman 3-9M	23	0	Single	Unemployed	High school
Pregnant Woman 4-2M	24	0	In a relationship	Employed part-time	High school
Pregnant Woman 5-2M	23	0	In a relationship	Employed part-time	High school
Pregnant Woman 6-2M	34	1	In a relationship	Unemployed	High school
Given Birth TR7	38	0	In a relationship	Employed full-time	High school
Pregnant Woman 8-6M	24	0	Married	Employed full-time	Matric
Pregnant Woman 9-6M	26	0	In a relationship	Self-employed	Primary
Pregnant Woman 10-7M	25	1	In a relationship	Unemployed	Primary
Pregnant Woman 11-8M	33	1	In a relationship	Unemployed	Matric
Pregnant Woman 12-3M	23	0	In a relationship	Unemployed	High school
Given Birth TR13	21	2	In a relationship	Unemployed	High school
Given Birth TR14	35	3	In a relationship	Unemployed	High school
Given Birth TR15	30	1	In a relationship	Unemployed	High school

#### Delay 1: Decision to seek care

The results from this study show that challenges that delayed women’s decision to seek antenatal care were unawareness of pregnancy, waiting for the baby bump to show, fear of HIV tests and blood withdrawals.

**Unaware of pregnancy:** Women expressed that they delayed booking early for antenatal care because they were unaware that they were pregnant. Inconsistent menstrual cycles were a confirmation for women that they were not pregnant. One woman reported that the challenge of not knowing she was pregnant prevented her from getting an abortion:

‘I was pregnant, but I was still having my period. That is why I did not go to the clinic because I did not know I was pregnant.’ (Pregnant Woman 11-8M)

‘I did not know at first. I found out when I was three months pregnant with my first child. I was unsure because I was getting my normal periods.’ (Pregnant Woman 3-9M)

‘I could not afford to have a second child. I tried to abort, but I was far too gone into the pregnancy and had no choice but to accept the child.’ (Pregnant Woman 8-6M)

**Waiting for the baby bump to show:** Pregnant women reported that they delayed booking antenatal care early because they were waiting for the baby bump to show. Waiting for the baby bump show was associated with cultural notions of keeping pregnancy a secret to protect the mother and the baby from evil spirits:

‘I am waiting for three months at least. The baby bump must show. I will go for sonar to check how many months, and then I will take it from there.’ (Pregnant Woman 4-2M)

‘I was relaxed because I was waiting for my baby bump to show. It only showed when I was seven months pregnant.’ (Pregnant Woman 1-8M)

‘Witchcraft is real. You cannot tell everyone you are pregnant before three months. Otherwise, people will attack you and the baby. That is why I did not go to the clinic early.’ (Given Birth TR15)

**Resentment of medical tests:** Pregnant women expressed that they delayed initiating antenatal care because they hated blood withdrawals and endless urine sample tests. Participants reported that clinicians do not explain the significance of these medical tests:

‘They draw too much blood from us without explaining why they are drawing it. Taking urine samples and blood pressure tests every time you go for an appointment at the clinic is tiring.’ (Pregnant Woman 3-9M)

Fear of getting tested for HIV was another challenge that delayed women from initiating antenatal care. Women reported that the psychological burden of dealing with an HIV diagnosis during pregnancy is too heavy. Women who had recently been diagnosed with HIV were reluctant to go back for antenatal care because the clinic was a painful reminder that they live with HIV:

‘I did not go because I hate being tested for HIV. At the clinic, you cannot even say no to the HIV test. I am pregnant, and I must deal with HIV. That is too much for me.’ (Pregnant Woman 6-2M)

‘You will be in the queue to check the baby, and then another nurse comes and says: follow me to a tent. That thing reminds you of your HIV status. You are fine when you are at home, but when you return to the clinic, it reminds you of your HIV status.’ (Pregnant Woman 8-6M)

#### Delay 2: Reaching the medical facility

The results from this study show that women delayed seeking care during pregnancy to reduce clinic visits because of the challenges associated with reaching the health facility. Long distance to travel to the clinic, transportation costs, inability to get time off work and the clinic demarcation system hindered women from reaching the health facility.

**Reduce clinic visits:** Women in this study reported that they deliberately booked late for antenatal care to reduce the number of clinic visits. Booking late for antenatal care was done to avoid challenges such as long distance to travel to the clinic and high cost of transportation fees. One woman reported that the clinic is far from where she works, and she cannot get leave days from work to attend antenatal care:

‘I did not want to book sooner because the clinic is a bit far from where I work and stay.’ (Given Birth TR7)

‘Money is tight these days. My boyfriend lost his job, and I cannot afford taxi fare to get to the clinic.’ (Pregnant Woman 1-8M)

‘The other thing is that at work, we do not get leave days easily. So, I decided to start attending the clinic properly next year.’ (Pregnant Woman 5-2M)

**Clinic demarcation system:** In South Africa, public health facilities are demarcated according to people’s physical addresses. This policy requires people to seek care in public health facilities closer to where they live. Pregnant women reported that they were turned away from antenatal care clinics outside their areas. Health care professionals told women to go back to clinics designated to their areas. Participants reported that this clinic clustering demarcation limits their options and discourages them from continuing with attending antenatal care:

‘Sometimes, when you go to the clinic, they will tell you that you must go to the clinic in your area. I am from Mofolo, and if I go to Dobsonville clinic, they will say that I must go back to Mofolo clinic because the clinic is closer to where I stay.’ (Pregnant Woman 6-2M)

‘Nurses transferred me to another clinic at Kibler Park, and it is far, and I do not have transport fare. So, it is challenging for me.’ (Pregnant Woman 10-7M)

‘They told me to go back and go to the relevant clinic. I used to go to the Mofolo clinic, and they said I must go to the Zola clinic because that is where I live. I do not like going to Zola Clinic. It is always packed.’ (Given Birth-TR13)

#### Delay 3: Receiving adequate treatment

Women in this study reported that structural challenges such as long waiting lines, shortage of equipment and essential medicines, deteriorating infrastructure, language barriers and poor attitude of nurses compromised the quality of treatment they received in public health facilities. Structural challenges in public health facilities discouraged women from returning for follow-up visits for antenatal care.

**Long waiting lines:** Pregnant women in this study reported that they deliberately booked late for antenatal care to avoid the long waiting lines they are subjected to in public clinics. Attending antenatal care after 6 months reduced the burden of long waiting lines. Women complained that nurses take time to attend to their needs and go on extended lunch breaks:

‘It is better to go when you are seven months pregnant because you will only wait in those long lines for one month. If you start going when you are two months pregnant, you will wait in those lines.’ (Pregnant Woman 6-2M)

‘Let us say I go to the clinic maybe around 06:00. You will be sitting there for hours. Nurses might stop the queue and say that they are going for lunch … and we will sit there for a long time.’ (Pregnant Woman 8-6M)

‘I do not even try going to the clinic. The line is long and does not move.’ (Given Birth TR7)

‘We get there at 06:00, and they only open the gate at 07:00. Nurses attend to us only at 09:00. That stopped me from going to the clinic because you wait and wait. The nurses do not want to work. You cannot arrive at the clinic at 06:00 and finish at 14:00. So I end up not going to the clinic because of that.’ (Pregnant Woman 3-9M)

**Shortage of equipment:** Shortage of medical equipment in public clinics discouraged women from returning for antenatal care follow-up visits. Participants shared that there is only one BP machine at the clinic to monitor high blood pressure for patients. Pregnant women must wait for hours to get their blood pressure checked. Women also reported that heart monitors are unavailable at the clinic to check the baby’s heart rate:

‘They only have one BP machine at the clinic. This machine is used by pregnant women and other departments that check sick people. I will be at the clinic by 05:00 and return home by 16:00 because of BP machine shortages.’ (Given Birth TR14)

‘There is a machine that checks the baby, not the sonar, the one with jelly. The heart rate machine is not there. That is something that bothers me because I will come back not knowing the heartbeat of the baby.’ (Pregnant Woman 1-8M)

**Shortage of essential medicines:** Shortage of essential medicines in public clinics discouraged pregnant women from returning for follow-up antenatal care visits. Pregnant women reported that it was useless to attend antenatal care because the clinic does not have medicines and they must buy it:

‘There is no folic acid in the clinic. They say you must buy it for yourself. So why must [*I*] go back there if they do not have medication?’ (Pregnant Woman 3-9M)

**Deteriorating infrastructure:** Deteriorating infrastructure in public health facilities hindered pregnant women from attending antenatal care. Pregnant women lamented that the toilets at the clinic are dirty, do not flush properly, and that there are no toilet papers. Broken toilets strain pregnant women because they pee frequently and are subjected to multiple urine tests:

‘Hygiene is important. We have one toilet at the clinic and share it. And there is no toilet paper.’ (Pregnant Woman 6-2M)

‘We must pee all the time because of the urine tests. And the toilets are broken.’ (Pregnant Woman 3-9M)

**Language barriers:** Women from other parts of Africa reported that they are reluctant to attend antenatal care because they struggled to converse with nurses. Women reported that nurses speak to them using South African languages they do not understand. Even if they tried to speak some South African languages, such as Shangan or Xitsonga, the nurses would tell them they could not converse in those native languages:

‘I am a foreigner here in South Africa, and I do not speak all the languages. So, I have noticed a language barrier, so if they could get someone to translate, that could be helpful.’ (Pregnant Woman 1-8M)

‘When you speak Shangan or Xitsonga, as it is known here in South Africa, they will tell you that they do not understand it. There is no point in going back there if they cannot hear what I am saying.’ (Pregnant Woman 9-6M)

**Poor attitudes of nurses:** In this study, poor treatment from nurses, such as disrespect, impatience, verbal abuse and unresponsiveness was highlighted as a challenge that discouraged women from returning for follow-up antenatal care visits. Women expressed that nurses do not care about their pregnancy and take long lunch breaks, extending their waiting time to receive care:

‘The nurses are impatient; they do not talk to you in a respectful manner. The treatment we get from clinics is bad.’ (Pregnant Woman 6-2M)

‘We do not go because of those nurses. I go to the clinic around 06:00 and I will be sitting there for hours, and then they come at you at their own time. And they shout at you.’ (Pregnant Woman 8-6M)

‘The treatment from the nurses is bad. They do not care about us or our babies. They just do things to pass the time. They talk too much and then go for their tea break before helping us.’ (Pregnant Woman 3-9M)

Women expressed that pregnancy is a critical stage characterised by uncertainty and anxiety. However, the nurses do not respond with adequate information when they ask for guidance about the baby’s movement and safety:

‘They dismiss your questions and tell you that “I must attend to other patients”. They do not check us or explain what is happening to our bodies. And you are left confused and scared.’ (Pregnant Woman 3-9M)

‘You know when you are pregnant people do not understand. If you go and say you are experiencing this in your body, and the baby is moving like this, the nurses look at you and say it is normal. And maybe you might go into labour and have a premature baby.’ (Pregnant Woman 8-6M)

Nurses were also accused of prescribing medication without explaining what it is for and why it must be taken. One woman mentioned that she is not attending antenatal care because she heard rumours that the medication they prescribe at the clinic makes pregnant women sick:

‘They just give us the medication and do not explain how often you must take it.’ (Pregnant Woman 1-8M)

‘I heard that the medication they give you during pregnancy can make you sick … So, I do not go to the clinic.’ (Pregnant Woman 4-2M)

## Discussion

The three-delay model was applied in this study to understand the challenges of women with antenatal care attendance in Soweto, Johannesburg. According to Thaddeus and Maine^35,36,37^, delay one, the decision to seek care during pregnancy is influenced by women’s educational level, socio-economic conditions, cultural beliefs, past pregnancy experiences and perceived barriers in reaching the health facility. Results from this study show that women’s decision to attend antenatal care was delayed because of challenges such as unawareness of pregnancy, waiting for the baby bump to show and fear of being tested for HIV. Women’s unawareness of pregnancy in this study must be read within a context where half of the participants were first-time mothers and with high school education, limiting their access to accurate information about reproduction. For women in Timor-Lester in Southeastern Asia, the contestation between medical knowledge of reproduction and indigenous knowledge of reproduction delayed the decision to seek care during pregnancy.^48^ In Timor-Lester, women waited for the baby to move as a confirmation of pregnancy.^48^ When the baby finally moves, the woman would consult with her husband and the older woman in her family, who grants her permission to seek medical care.^48^ Similar studies were observed by Kaiser^49^ in Zambia where the woman’s decision to seek care during pregnancy depends on permission from the husband, mother-in-law and older women in the family.

Waiting for the baby bump to show in this study was associated with cultural notions of keeping pregnancy a secret because of possible spiritual attacks from the public that may endanger the baby. The cultural notion of keeping pregnancy a secret was also observed by Mulondo et al.^31^ in Limpopo, where women were not supposed to disclose their pregnancy before 3 months, as doing so would expose them to witchcraft that could kill the baby. A study conducted by Sibiya et al.^50^ in eThekwini District in KwaZulu-Natal, women reported that they are culturally prohibited from attending antenatal care early as publicising pregnancy in the community can lead to spontaneous miscarriages. Women in this study delayed initiating antenatal care because they feared being tested for HIV. An HIV diagnosis during pregnancy was reported as too much of a burden to bear. In South Africa, the HIV Prevention-of-Mother-to-Child (PMTC) programme has managed to detect and treat maternal HIV infections to ensure that unborn babies are not exposed to HIV.^51^ However, the downside of PMTC programme is that it exposes women to the psychological burden of dealing with an HIV status while pregnant.^51,52,53,54^ Fear of testing for HIV during pregnancy was also observed in a study conducted by Brittain Myer et al.^55^ in Cape Town, who found that pregnant women are afraid of taking Antiretrovirals (ARVs) because they will be forced to disclose their HIV status to their partners. This has led to cases of gender-based violence where pregnant women are blamed and beaten up for bringing HIV into the relationship.^55,56,57^

Delay two are challenges that a pregnant woman and her family face when trying to reach the health facility, such as the conditions of roads, availability and affordability of transportation and distribution of health facilities.^38,39,40^ The results from this study show that women deliberately delayed attending antenatal care because of long distances to travel to the clinic, transportation costs, inability to get time off work and the clinic demarcation system. Women’s inability to afford transportation costs to reach the clinic needs to be contextualised, where nine out of 14 participants in this study were unemployed and had no source of income. Murewanhema et al.^28^ also found that in sub-Saharan Africa, women delay antenatal care services because of the physical strain and financial burden of high transportation fees that come with getting to health facilities. In a study conducted by Kaswa et al.^15^ in Embekweni in the Eastern Cape, women reported that they did not attend antenatal care because they had to travel long distances by foot to get to the clinic. A study conducted in Malawi by Combs Thorsen^58^ found that poor road infrastructures, long distances to the hospital and uneven distribution of health facilities resulted in women with obstetric complications dying before they reached the hospital. Similar findings were observed by Paswan et al.^59^ that pregnant women in rural parts of India struggle to reach medical health facilities because of muddy roads during the rainy season.

When the pregnant woman finally reaches the health facility, she can experience delay three, which is the provision of adequate care which depends on the availability of skilled health care workers, medical suppliers, medicines and efficient referral systems.^40,41^ Results from this study show that long waiting lines, shortage of equipment and essential medicines, deteriorating infrastructure in public facilities, language barriers and poor attitudes of nurses compromised the quality of treatment women received during antenatal care visits. A study conducted by Solarin and Black^60^ in Central Johannesburg found that women resented attending antenatal care because of long waiting lines, where only one nurse was responsible for attending to more than 50 pregnant women a day. Nurses reported that some days, they must tell the pregnant women to return the following day because the clinic ran out of essential medicines and medical testing kits.^60^ According to van Pelt et al.,^61^ in Tanzania, health care workers reported there is nothing they can do for pregnant women when there are not enough HIV kits, medicines and vaccines.

Language is critical to patient optimal care experience and can compromise the quality of health care delivery and patient safety.^62^ A study conducted by Hunter-Adams et al.^63^ found that pregnant women from Zimbabwe could not speak the local South African language (IsiXhosa), leading to being labelled and stereotyped by health care workers. Poor attitudes of nurses and their ill-treatment of pregnant women in public health facilities in South Africa can be characterised as obstetric violence.^64^ Pregnant women are subjected to dehumanising treatment that ranges from verbal insults to discrimination and intolerance.^65^ However, Govender et al.^66^ warn that nurses’ insult, discrimination and intolerance towards pregnant women must be read in a context where nurses practise in dysfunctional public health facilities. Nurses in public facilities face difficult working conditions such as lack of equipment and medication, patient overload, underpayment, and burnout.^67,68,69,70,71,72^

## Conclusion

From this study, we learn that addressing challenges that hinder pregnant women from accessing antenatal care services is necessary and urgent to eliminate the burden of maternal and infant mortality in South Africa. A comprehensive reproductive health education strategy that will teach pregnant women, families and communities about the importance of antenatal care must be implemented. This reproductive health education strategy must take cultural notions of pregnancy seriously among different communities in the country. The PMTC programme in antenatal care clinics must include effective continuous psychological counselling services to assist pregnant women dealing with the psychological burden of an HIV diagnosis. Integrating antenatal care services with community-based health services is necessary, and health care professionals, with the help of community health care workers, can deliver services to pregnant women in the comfort of their homes. There is an urgent need for health care system reforms to address shortages of health care facilities, health human resources, essential medicine and equipment to improve the quality of antenatal care services in health facilities. The limitation of this study is that it was conducted among 10 pregnant women and four women who had recently given birth in Soweto, Johannesburg, and these qualitative findings cannot be generalised to other contexts. Another limitation of this study is that it did not engage with key members of the family such as husbands, partners and relatives to assess their role in influencing women’s decisions to attend antenatal care. Future studies can interrogate how actors within the family structure and the community influence pregnant women’s decision to attend antenatal care in Soweto, Johannesburg.
